# Migration, Acculturation and Environment: Determinants of Obesity among Iranian Migrants in Australia

**DOI:** 10.3390/ijerph120201083

**Published:** 2015-01-22

**Authors:** Maryam Delavari, Anders Larrabee Sønderlund, David Mellor, Mohammadreza Mohebbi, Boyd Swinburn

**Affiliations:** 1 WHO Collaborating Centre for Obesity Prevention, Faculty of Health, Deakin University, Melbourne, Victoria 3125, Australia; 2 School of Psychology, College of Life and Environmental Sciences, University of Exeter, Exeter EX4 4QJ, UK; E-Mail: a.l.sonderlund@exeter.ac.uk; 3 School of Psychology, Faculty of Health, Deakin University, Melbourne, Victoria 3125, Australia; E-Mail: david.mellor@deakin.edu.au; 4 Biostatistics Unit, Faculty of Health, Deakin University, Melbourne, Victoria 3125, Australia; E-Mail: m.mohebbi@deakin.edu.au; 5 School of Population Health, University of Auckland, Auckland 1142, New Zealand; E-Mail: boyd.swinburn@auckland.ac.nz

**Keywords:** acculturation, obesity, physical environment, immigration, health, Iranians

## Abstract

While migration from low- to high-income countries is typically associated with weight gain, the obesity risks of migration from middle-income countries are less certain. In addition to changes in behaviours and cultural orientation upon migration, analyses of changes in environments are needed to explain post-migration risks for obesity. The present study examines the interaction between obesity-related environmental factors and the pattern of migrant acculturation in a sample of 152 Iranian immigrants in Victoria, Australia. Weight measurements, demographics, physical activity levels and diet habits were also surveyed. The pattern of acculturation (relative integration, assimilation, separation or marginalization) was not related to body mass index, diet, or physical activity behaviours. Three relevant aspects of participants’ perception of the Australian environment (physically active environments, social pressure to be fit, unhealthy food environments) varied considerably by demographic characteristics, but only one (physically active environments) was related to a pattern of acculturation (assimilation). Overall, this research highlighted a number of key relationships between acculturation and obesity-related environments and behaviours for our study sample. Theoretical models on migration, culture and obesity need to include environmental factors.

## 1. Introduction

The shift towards high-energy diets and sedentary life styles has contributed to increasing global obesity rates over the last three decades [1,2]. This trend is associated with serious non-communicable diseases such as diabetes and heart disease [3,4,5] which now dominate the global burden of illness. The World Health Organization (WHO) recommends prevention strategies, such as public health education campaigns and health promotion programs, to fight obesity [6,7]. The success of such interventions depends on the extent to which the programs and messages resonate with the audience, and thus audience characteristics are highly relevant within this context. With countries becoming increasingly multicultural through migration, the resulting wide variation in ethnic, cultural, religious and demographic backgrounds needs to be considered in the development and implementation of health promotion programs and campaigns [1,8,9,10,11]. 

Many Western countries are experiencing an increase in obesity prevalence, and many of them also have substantial migrant populations [8]. What is the experience of migrants in terms of weight change as they move from one country to another? Most of the research on this has been conducted in high-income countries with migrants from low-income countries [8]. Migrant groups in Australia are a good case in point [8,12]. A number of studies have reported that migrants to Australia often experience weight gain following migration, and record even higher body weights than their local counterparts after a typical length of residency of six months to 10 years [13,14,15,16,17]. This experience particularly relates to immigrants from countries with a low or medium human developmental index (HDI) to countries with a high HDI [18]. Moreover, there are differences in obesity rates between various groups of migrants, and this may depend on factors other than migration, including for example, ethnicity [19], age at time of migration, length of residency in the new country [20], and acculturation pattern [8]. 

For immigrants, the pattern of acculturation is associated with the interchange of attitudes, values and behavioral patterns between the culture of origin and the host culture. Importantly, these changes may relate to health behaviors, including those associated with obesity [8]. A range of studies, however, shows that the relationship between obesity and acculturation patterns differs between particular immigrant groups. For example, Fitzgerald, *et al.* [21] demonstrated an overall positive correlation between the degree of acculturation and body weight in Mexican-Americans—a finding which has been supported in other studies [8]. In contrast, while Lee *et al.* [22] found a significant relationship between the degree of acculturation and obesity in their male sub-sample, no such relationship was evident for their female sub-sample, suggesting gender differences in the association between body mass index (BMI) and acculturation. Other studies again have found negative or mixed associations, indicating less obesity in migrant women than men [23,24]. These results emphasize the notion that, in all likelihood, a complex interaction between culture, ethnicity, host country and place of origin impacts on a range of health-related factors, including obesity. Thus, an area of research that could help clarify the relationship between body weight and migration relate to the change from the home to the host environments. The inclusion of these environmental factors is generally lacking in the literature, and migrant populations who move between countries with similar obesity rates, but dissimilar home environments, need further exploration. 

Environments are defined as the sum of influences of surroundings, opportunities, or conditions of life [25]. Dissecting this further, Swinburn *et al*. [26] divide obesogenic environments into two levels (micro/settings and macro/sectors) and four types of environments (*physical* “what is available?”, *economic* “what are the costs?”, *policy* “what are the rules?” and *socio-cultural* “what are the values, beliefs, and attitudes?”) [25]. Thus, a key question concerns the extent to which differences between home and host environments influence migrants’ body weight change. While there is some research touching on this [25], there are no studies specifically investigating how these environmental factors interact with patterns of acculturation to explain health-related outcomes, including obesity rates. Indeed, theories and constructs about how migration influences perceptions, behaviors and weight change, need to be expanded to incorporate the changes experienced by migrants moving from home to host environments. 

Iranians represent a unique and emerging migrant group in Australia. They are generally well educated and come from a medium HDI country that has an obesity pattern similar to high HDI countries (including Australia). They tend to immigrate for reasons related to personal freedom as opposed to economic and material disadvantages, and emigrate from a complex religious/political environment. Thus, studying Iranian migrants to Australia may provide insights into the interactions between migration, environments, culture and obesity.

### Current Study

The present study investigated the relationship between the pattern of acculturation and a number of environmental factors, and particularly how these factors might be associated with obesity in a sample of Iranian immigrants in Victoria, Australia. While this is a cross-sectional study, the unique features of Iranian migration to Australia, and the specific inclusion of environmental factors, may help to inform the models and theories of acculturation and obesity.

## 2. Method 

### 2.1. Study Design and Participants

A cross-sectional study design was used and included a convenience sample of 152 Iranian migrants living in Victoria, Australia. The recruitment method used included broad advertising of the study and word of mouth. The study was advertised with the assistance of various Iranian communities, including the Iranian Students of Victoria Alumni (ISVA), the Iranian Society of Victoria (ISOV), and the Iranian Persian Language Weekend School in Melbourne. Through these institutions it was possible to reach guardians and parents of current students. Further, study flyers and posters were put up at Iranian shops. The inclusion criteria for study participation stated that participants had to (i) have been born in Iran; (ii) be between the ages of 18 and 65 years, and; (iii) have lived in Australia for at least 6 months (we chose this period to ensure that participants had settled in the new environment and could report on their settled daily life in Australia). Once prospective participants signed up for the study, they were asked to invite friends and acquaintances whom they believed met the inclusion criteria. Initially, 200 individuals were recruited for the study. Of these, 152 individuals completed the study. All subjects gave their informed consent for inclusion before they participated in the study. The study was conducted in accordance with the Declaration of Helsinki, and the protocol was approved by the Deakin University Human Research Ethics Committee (DUHREC), approval number HEAG-H 90/2011.

### 2.2. Study Measures

Data collection included measurement of height (cm), weight (kg) and waist circumference (cm) as well as the administration of four questionnaires designed to assess: (i) participant demography, (ii) food habits and physical activity, (iii) pattern of acculturation, and finally, (iv) the experience of environmental determinants of obesity in Australia. 

The socio-demographic questionnaire was adapted from the key demographic items used in health surveys of the Australian Institute of Health and Welfare. Food habits were measured with a 10-item food frequency questionnaire adopted from the Nutrition Survey User’s Guide [26] and the physical activity questionnaire was adapted from the Active Australia Survey [27]. To assess acculturation, we chose a bi-dimensional acculturation scale, the Vancouver Index of Acculturation (VIA) [28]. The VIA is a self-report measure that has been demonstrated as a valid and reliable scale to be used among migrant groups, including Middle Eastern and Muslim migrants in high-income countries such as Australia [29,30]. The scale comprises 20 items which focus on several domains relevant to acculturation, including values, social relationships, and adherence to tradition. Items include for example *“I often participate in my heritage cultural traditions”*, *“I would be willing to marry a white Australian person”*, *“I often behave in ways that are typically Australian”*, *“It is important for me to maintain or develop the practices of my heritage culture”*. Participants completed the same set of questions for both their traditional and their new culture [18,21]. In line with Berry [31], acculturation patterns were determined by dividing participant responses on the VIA subscales (*i.e.*, *Iranian-orientation* and *Australian-orientation*) into those above (high) and below (low) the mean. Participants were grouped in four categories of relative cultural orientation. Participants who scored low on both the *Iranian-orientation* and *Australian-orientation* subscales, and thus were relatively alienated from both Iranian and Australian cultural orientation, were termed as *marginalized* migrants. Those participants with relatively high scores on both the *Iranian-orientation* and *Australian-orientation* subscales were referred to as *integrated* migrants. Those participants with low scores on the *Iranian-orientation* and high scores on the *Australian-orientation* subscales were considered to be *assimilated* migrants, identifying more with Australian culture than their original Iranian culture. Those participants who scored low on *Australian-orientation* and high on *Iranian-orientation* were categorized as *separated* migrants, as they preferred their original Iranian heritage over the new Australian culture. 

Finally, the Migrant Obesogenic Perception of the Environment questionnaire (MOPE-Q) [25] was used to gauge the experience of environmental determinants of obesity. The MOPE-Q was specifically developed for the measurement of the influence of environments on migrant obesity and has been demonstrated to be both valid and reliable in this context [25]. This questionnaire is a 17-item scale designed to measure the environmental determinants of obesity that migrants might experience in Australia. Each item is rated on a 7-point Likert scale, ranging from strongly disagree (1) to strongly agree (7) [25]. The scale comprises four environmental subscales for Australia (*i.e.*, Physically Active Environments, *Social Pressure to be Fit*, *Unhealthy Food Environments*, and *Government Livability Policies*). The first subscale measures how the availability of green spaces, walking and cycling paths as well as the culture of physical activity in Australia may encourage Iranian migrants to be physically active. The second subscale, *Social Pressure to be Fit* consists of five items which measure how the routine socio-cultural pressure to be fit and conscious of body size, as well as people’s negative attitudes about overweight people, might pressure migrants to be concerned about their weight. The third subscale, *Unhealthy Food Environment* relates to the obesogenic food environment in Australia (*i.e.*, ready access to unhealthy food service outlets, cheap unhealthy food and the high level of advertising for unhealthy foods) that could encourage Iranian migrants to have unhealthy food habits. Finally, the fourth subscale, *Government Livability Policies* includes four items pertaining to how the Australian government’s “livability” policies may encourage Iranian migrants to be physically active and concerned about their weight (e.g., media health campaigns may encourage physical activity). 

### 2.3. Procedure

Data collection occurred in August and September, 2012. Informed consent was obtained from participants, all of whom volunteered after reading the plain language statement describing the study. To ensure maximum accuracy of body measurements, a trained research assistant undertook this task for each participant. Measurements were made twice and if the difference between the first and the second measurement were equal or greater than 5 mm for height, 10 mm for waist circumference and 0.1 kg for weight, a third measurement was taken. The questionnaires took approximately 45 min to complete. The data collection instruments and procedures were undertaken in English. However, any difficulties experienced by participants in understanding the questions were addressed by use of bi-lingual (Farsi and English) research assistants who were hired and trained for the research project to assist with interpretation and translation. At the end of the survey administration, each participant received a movie ticket voucher as a token of appreciation. 

### 2.4. Data Analysis

**Figure 1:**
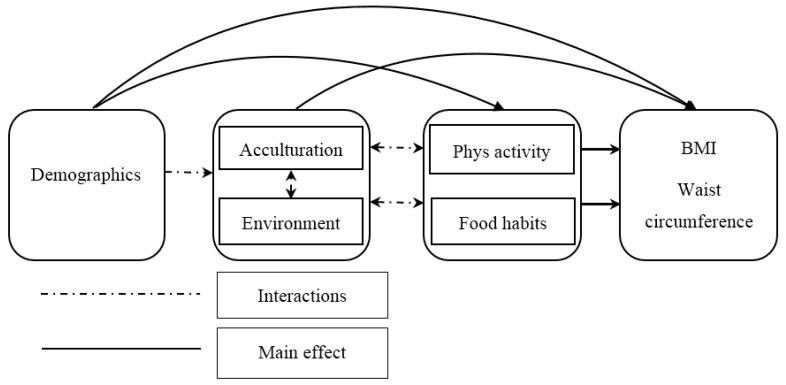
Path diagram for data analysis.

Analysis of the data was conducted in two stages (Figure 1). For the first stage, exploratory correlation coefficients, one-way analyses of variance and chi-square tests were carried out to get an initial idea of any correlations and mean differences in the data. In the next stage, multiple linear regression analyses were conducted with acculturation (VIA) and environmental (MOPE-Q) variables as the principal outcome measures. A two-step procedure was implemented in variable selection for multiple linear regression modeling. In the first step, univariate analysis was conducted. Multivariate linear regression was then performed to include all variables associated with the environmental factors or acculturation patterns at *p* ≤ 0.1 (see Table 1). 

**Table 1:** Demographic, behavioural (physical activity and diet) and anthropometric characteristics by acculturation categories.

Characteristic	Integration	Assimilation	Separation	Marginalisation	Statistics
	N (%)	N (%)	N (%)	N (%)	
***Total sample***	39 (25.8)	33 (21.9)	31 (20.5)	48 (31.8)	
***Gender***					
Female	24 (29)	19 (57.6)	14 (45.2)	26 (54.2)	*X^2^* = 1.99
Male	15 (38.5)	14 (42.4)	17 (54.8)	22 (54.8)	*p* = 0.58
***Education***					
Tertiary	34 (87.2)	33 (100)	30 (96.8)	44 (91.7)	*X^2^* = 6.77
Primary and secondary	5 (12.8)	0 (0.0)	1 (3.2)	4 (8.3)	*p* = 0.34
***Marital status***					
Married	29 (74.4)	22 (66.7)	22 (70.9)	26 (54.2)	*X^2^* = 4.52
Single	10 (25.6)	11 (33.3)	9 (29.1)	22 (45.8)	*p* = 0.21
***Religion***					
Islam	27 (69.3)	18 (54.5)	20 (64.5)	36 (75)	*X^2^* = 8.29 *p* = 0.21
Other religion	3 (7.7)	5 (15.2)	1 (3.3)	6 (12.5)	
No religion	9 (23)	10 (30.3)	10 (32.2)	6 (12.5)	
***Region of residency prior to migration***					
Capital city	36 (92.3)	30 (90.1)	24 (77.5)	44 (91.7)	*X^2^* = 11.13
Others (small city or rural communities)	3 (7.7)	3 (9.9)	7 (22.5)	4 (8.3)	*p* = 0.26
***Reason for migration***					
Family reunion	4 (10.3)	1 (3.0)	2 (6.5)	8 (16.7)	*X^2^* = 25.87 *p* = 0.00
Education	10 (25.7)	17 (51.5)	18 (58.1)	27 (56.2)	
Work	6 (15.4)	10 (30.3)	4 (12.9)	5 (10.4)	
Others (including political upheaval)	19 (48.6)	5 (15.2)	7 (22.5)	8 (16.7)	
***Net income***					
≥ $1800	10 (25.6)	10 (30.3)	3 (9.7)	4 (8.3)	*X^2^* = 15.93 *p* = 0.07
$600–$1799	14 (35.9)	6 (18.1)	10 (32.2)	11 (22.9)	
$1–$599	11 (28.2)	9 (27.2)	8 (25.8)	19 (39.6)	
Not applicable	4 (10.3)	8 (24.4)	10 (32.3)	10 (29.2)	
***Employment status***					
Employed, full-time	15 (38.5)	10 (30.3)	6 (19.4)	7 (14.6)	*X^2^* = 12.64 *p* = 0.18
Employed, part-time	7 (17.9)	2 (6.1)	4 (12.9)	9 (18.7)	
Student	10 (25.6)	17 (51.5)	15 (48.4)	23 (47.9)	
None of the above	7 (18.0)	4 (12.1)	6 (19.3)	9 (18.8)	
***Physical activity***					
Active	15 (28.5)	18 (54.5)	12 (38.7)	20 (41.7)	*X^2^* = 2.39
Inactive	24 (61.5)	15 (45.5)	19 (61.3)	28 (58.3)	*p* = 0.49
***Food habits***					
Healthy	85 (53.8)	21 (51.5)	21 (67.7)	26 (54.2)	*X^2^* = 2.14
Unhealthy	66 (46.2)	10 (48.5)	18 (32.3)	22 (45.8)	*p* = 0.55
	**Mean (SE)**	**Mean (SE)**	**Mean (SE)**	**Mean (SE)**	
***Age (year)***	38.8 (10.9)	32.0 (8.4)	34.9 (9.0)	33.0 (9.5)	*p* = 0.01
***Body mass index (kg/cm^2^)***	25.1 (3.3)	24.8 (4.1)	25.1 (3.9)	25.3 (3.6)	*p* = 0.95
***Waist circumference (cm)***	86.3 (8.8)	84.0 (13.7)	86.3 (12.5)	87.1 (11.9)	*p* = 0.68
***Length of residency in Australia (year)***	5.2 (5.8)	4.3 (5.6)	4.26 (4.9)	2.77 (3.8)	*p* = 0.15

Multinomial logistic regression models were used, reporting adjusted odds ratios and corresponding 95% confidence intervals, to adjust for any important covariates (*i.e.*, *p* ≤ 0.1) in evaluating the relationships between the acculturation pattern and BMI or waist circumference. A backward elimination method with exclusion criteria of 0.1 was used for model variable selection. Spare cells from univariate tables were collapsed to obtain a robust result in the multivariate analysis. Each of the four subscales of the environmental MOPE-Q survey were examined separately as a dependent variable with the other three as independent variables. 

## 3. Results

Males comprised 55.3% (*n* = 84) of the total sample, and females 44.7% (*n* = 67). The age-range was 19 years to 65 years, though most were in their late 20s and 30s, resulting in a mean age of 34 years. The time that had passed since immigration to Australia was between six months and 28 years, with the average being approximately four years. The reasons for migration were *education* (48%, *n* = 73), *political upheaval* (25.7%, *n* = 38), *employment* (16.4%, *n* = 25), and *family reunion* (9.9%, *n* = 15).

### 3.1. Acculturation

**Figure 2:**
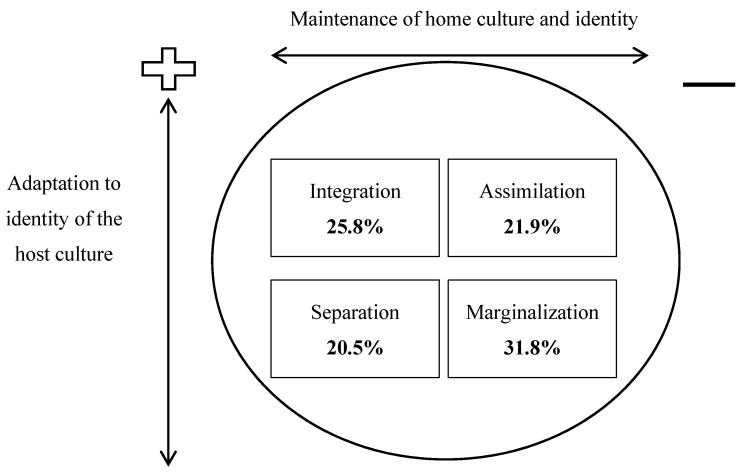
Relative categories of cultural orientation of participants.

Overall, according to the VIA, the participants were more inclined to the *Iranian-orientation* (M = 52.7, SD = 9.7) than the *Australian-orientation* (M = 44.9, SD = 8.8). Acculturation category was determined by dividing participant responses on the subscales (*i.e.*, *Iranian-orientation* and *Australian-orientation*) into those above (high) and below (low) the mean, thus placing them into one of four categories of cultural orientation as described above and shown in Figure 2. 

In the first stage of the analysis we analyzed demographic characteristics by acculturation category (Table 1). There were significant relationships between acculturation category and both age (*p* = 0.010) and reason for migration (*p* = 0.002). Specifically, those categorized as *assimilated* to the Australian culture were younger, while those who were *integrated* were older participants. In regards to reason for migration, people in the *marginalized* category were inclined to have come to Australia for either family reunion or education (53.3% and 37.5% respectively). People who were categorized as *assimilated* were likely to have come for work (40%), while *integrated* people most often had migrated because of political upheaval in their country of origin (48.7%) (Table 1). Of note, there were no significant differences in BMI, waist circumference, physical activity, or diet across the four acculturation categories.

In the second stage of the analysis, multinomial logistic regression analyses were conducted. We examined adjusted odds ratios and corresponding 95% confidence intervals to control for any important covariates (e.g., age, gender, reason for migration, religion) in the relationship between the *acculturation categories* and the main dependent variables *BMI*, *waist circumference*, *food habits*, *physical activity*.

Multivariate linear regression models were also performed to evaluate the association between environmental factors (as outcomes) with acculturation patterns, food habits and physical activity (as independent factors) and demographic and socio-economic factors (as covariates) at *p* ≤ 0.1 (see Figure 1). When significant and marginally significant (*i.e.*, *p* < 0.05 and *p* ≤ 0.10) variables from the univariate analyses were included in the multivariate analysis, both age and reason for migration remained statistically significant with *p* < 0.05. 

### 3.2. Environment

Analyses of variance were conducted to test the relationships between environmental factors and a variety of independent variables (as listed in Table 2). Table 2 shows the relationships that were statistically significant or marginally statistically significant. The longer participants had been in Australia, the more they perceived and engaged with Australia as a physically active environment. In terms of reasons for migration, those who came for the sake of education experienced Australia more as a physically active environment compared to those who came for family reunion, work and other reasons (*p* = 0.04). Our study also revealed a significant association between the pattern of acculturation and participants’ experience of Australia as a physically active environment. Specifically, *assimilated* migrants experienced Australia more as a physically active environment compared to the three other acculturation groups (*p* = 0.00). 

**Table 2:** ANOVA results—the association between environmental factors and independent covariates.

Australian Environment Factor *	Independent Variable	Mean (SD) **	*p-value*
**Physically active environments**	**Reason for migration**		0.04
	Family reunion	26.46 (5.7)	
	Education	29.33 (4.3)	
	Work	26.96 (3.7)	
	Others ^a^	27.10 (6.2)	
	**Household income**		0.08
	≥$1800	27.70 (5.9)	
	$600–$1799	28.11 (6.1)	
	$1–$599	28.53 (4.2)	
	Not applicable	29.94 (4.1)	
	**The degree of acculturation**		0.001
	Integration	25.83 (6.5)	
	Marginalisation	29.83 (4.2)	
	Assimilation	29.88 (2.1)	
	Separation	28.58 (4.6)	
**Social pressure to be fit**	**Religion**		0.001
	Islam	20.43 (6.3)	
	Other religions	17.60 (6.9)	
	No religion	19.71 (6.7)	
	Marital status		0.06
	Married	20.57 (6.3)	
	Single	18.89 (6.7)	
	**Region of residency prior to migration**		0.04
	Capital city	19.55 (6.4)	
	Small city	24.40 (6.3)	
	Rural community	16.00 (0.0)	
	Not applicable	16.00 (0.0)	
**Unhealthy food environments**	**Income**		0.001
	≥$1800	12.19 (4.8)	
	$600–$1799	12.25 (4.8)	
	$1–$599	12.15 (5.2)	
	Not applicable	12.18 (5.2)	

* Based on 7-likert scale; ** Mean (SD) for the environmental factors for each category of the independent variables; **^a^** e.g., political asylum, refugee, *etc*.

Religion (*p* = 0.00) and region of residency prior to migration (*p* = 0.04) were significantly related to feelings of social pressure to be fit in Australia, with Muslims and participants from smaller cities in Iran more likely to feel this pressure. 

Links between unhealthy food environments in Australia and obesity-related covariates were also revealed. Among them, household income was the only significant factor (*p* = 0.00). Participants with middle income experienced Australia as a more unhealthy food environment compared to participants with higher or lower income. Finally, the relationships between government livability policy in Australia and obesity related covariates were assessed. None of the variables demonstrated a significant link to government livability policy. 

Since the ANOVA was significant for the association between acculturation status and the *Physically Active Environments* factor, pairwise comparisons with Bonferroni post-hoc adjustments were performed. Results showed significant differences between the *integration* and *assimilation* groups (*p* = 0.003) as well as *integration* and *marginalisation* groups (*p* = 0.001). 

Age and SES did not differ across groups and it was therefore not necessary to control for these variables in the analysis.

Linear regression models were implemented to evaluate association between Australian environment subscales (*i.e.*, *Physically Active Environments*, *Social Pressure to be Fit*, *Unhealthy Food Environments* and *Government Livability Policies*) pattern of acculturation, and covariates (*i.e.*, demographics if *p* ≤ 0.10 on univariate analyses). The *Physically Active Environments* subscale retained its significant correlation with the pattern of acculturation (*p* < 0.001), with *assimilated* Iranian-Australians (as opposed to the three other acculturation groups) viewing Australia as an environment conducive to physical activity. 

None of the other variables were associated with the Australian environmental subscales linked to obesity. In regards to the *Social Pressure to be Fit* subscale, none of the covariates remained statistically significant in the multiple regression model.

## 4. Discussion

**Figure 3:**
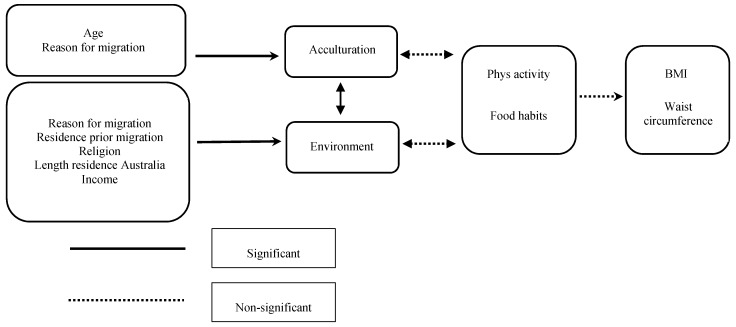
Path diagram showing the outcome of the path analysis. No significant interactions were found (see Table 1 and Table 2 for statistics).

This study aimed to investigate the association between acculturation, Australian environmental domains and obesity in a sample of Iranian immigrants to Australia. Importantly, results indicated that the pattern of acculturation did not relate to Iranian-Australian BMI or waist circumference. However, the pattern of acculturation was linked to the migrant perception and use of the Australian environment for health and activity. In particular, the assimilated (as opposed to marginalized, integrated or separated) participants were inclined to view and engage the Australian environment as one conducive to physical activity. Other factors affecting the migrant experience of the Australian environment included reason for migration, religion, income and region of residence in country of origin (see Figure 3). Specifically, migrants were more likely to perceive and use the Australian environment as a physically active one if they had migrated for education rather than for family or work, were Muslim or originally from a smaller town or city in Iran, or earned a relatively low or high household income (as opposed to a middle income). Thus, the present research provides the first evidence that acculturation pattern does not impact directly on BMI or waist circumference. Rather, acculturation pattern is, along with migrant background and socio-economic characteristics, related to migrants’ perception of the Australian environment as one conducive to health and fitness. 

The relationship between the degree of acculturation and body weight has been explored previously and has generally been more oriented to the host culture and linked to higher BMI [8,21]. However, any potential mediators and moderators of this relationship have not been explored closely. Of considerable importance given the context of the present study, the results showed a significant association between the pattern of acculturation and participants’ experience of Australia as an environment conducive to physical activity. Thus, assimilated participants were more likely to perceive and experience the Australian environment in terms of physical fitness and activity. Assimilated migrants may thus be more likely to engage with and embrace the physical environment and the Australian cultural identity of, for example, being a “sporting nation” [32]. In this way, this may create a situation in which a social and physical environment is experienced as conducive to physical activity may actually increase levels of physical exercise, and in turn protect against weight gain and high BMI (although these latter relationships were not detected in this study). 

In terms of research methodology and theory, these findings also provide extra insight. Specifically, they broaden the understanding of the relationship between migrant health and acculturation beyond cultural, tradition- and ethnicity-related factors to include those of the physical, economic, policy and socio-cultural environment to which people migrate. Most research to date has focused on the impact of the change in food and cultural environment (fast food culture, increased availability of cheap high-fat, high-sugar foods) and how these variables fit together. Our results, however, show that physical environment and the perception of that environment also play an important role, and one which should be included when considering the health effects of migration. However, the underlying mechanisms which facilitate this relationship are in reality quite complex. For example, we found significant differences between Iranian and Australian environmental factors linked to obesity, but these did not correlate with participants’ food habits, physical activity pattern and BMI. This speaks to the complexity of factors contributing to weight change. As our study demonstrates, the *pattern* of acculturation as well as various environmental factors interplay to both put migrants at risk of and protect them from the negative health effects associated with migration, including obesity. This, in turn, lends some credence to use of the Migrant Obesogenic Perception of the Environment questionnaire (MOPE-Q) [25] and its use in gauging more inclusively and comprehensively the impact of migration on migrant health as well as informing potential intervention. 

### Strengths and Limitations

There are several notable strengths of the current study. First, the broad scope of factors investigated allowed for an inclusive and comprehensive analysis of the relationship between obesity, acculturation and environment. Second, the measurement tools used for data collection (*i.e.*, the VIA and the MOPE-Q) have been demonstrated as robust, valid and reliable. Third, the particular use of Iranian migrant samples represent a different and potentially insightful case for testing migration and health theories, as Iranian migrants generally are comparable to Australians in terms of income, education, and BMI. 

The study was not without its limitations, however. The sample comprised only Iranian immigrants to Australia. Given the diversity of the immigrant population in Australia in terms of nationality, ethnicity, culture and SES, it can only be assumed that various immigrant groups respond differently to the Australian culture and environment as well as to the experience of migration in and of itself. Thus, further research with more ethnically, nationally and socio-economically inclusive samples is needed to properly understand the health effects of migration to Australia. 

An additional sampling limitation of the study concerned the possibility that the convenience sample design may have led to an over-representation of Iranians who were more integrated or assimilated than the general Iranian population in Australia. This may have affected the categorization of participants by acculturation as the mean score of the sample was used to derive categories rather than standardised cut-offs independent of the sample characteristics. Thus, for example, the *marginalised* group was marginalised relative to the rest of the participants, but the degree to which they were marginalized relative to the general Iranian population in Australia is not known. Further, not only did the veracity of much of the data depend on participant memory, but the lack of access to participants’ anthropometric measurements at the time of migration also did not allow us to observe the changes that happened in their BMI and waist circumference status since arrival in Australia. Future longitudinal research is needed to look at the interaction between pattern of acculturation and obesity related environmental factors on migrants’ weight over time.

Finally, the correlational nature of the regression analyses undertaken prohibits any assessment of cause and effect. Again, longitudinal or other quasi-experimental research designs might help gain further insight into the exact mechanisms and triggers underlying the relationship between migration and obesity. For example, Negy, Schwartz and Reig-Ferrer [32] found a relationship between violated expectations and acculturation stress in Hispanic immigrants in the U.S.. Thus, an interesting area for future research might focus on the extent to which immigrants’ experiences in their host country are dissonant to what the immigrants expected before arrival and how this impacts on the relationship between acculturation stress and weight gain.

## 5. Conclusions 

We showed the utility of combining environmental and acculturation measurement scales for understanding the determinants of overweight and obesity among migrant groups in Australia. We also briefly discussed the relationship between the pattern of acculturation and environmental factors in terms of obesity status and its determinants, and found that assimilated Iranian migrants were more likely to accept and endorse the Australian culture of physical activity. Thus, physical environment appears to contribute to the relationship between the pattern of acculturation and obesity in migrant populations. This expands on previous literature that focuses solely on acculturation (often using a uni-dimensional scale) and its impact on migrant obesity. These findings highlight the need for future prevention programs in Australia to be based on an understanding of the multiple environmental factors, including socio-cultural factors, which contribute to the experiences of migrants. Obviously, there is no “one-size-fits-all” solution to this problem, and thus, it will require collaborative work among various professions and the migrant communities themselves. Particularly in regards to policy makers, this study provides evidence-based information on community-level obesity risk-factors specific to immigrants. This evidence may inform future research and prevention efforts to curb obesity and overweight in such populations, and ultimately calls for public health and environment planning professionals and/or researchers to work together to combat the rising obesity epidemic in multi-ethnic countries like Australia. 
